# Multiple Negative Differential Resistances in Nanofluidic Conical Pores: A Phenomenological Model

**DOI:** 10.1021/acs.jpclett.5c03327

**Published:** 2025-11-19

**Authors:** Javier Cervera, Patricio Ramirez, Sergio Portillo, Saima Nasir, Mubarak Ali, Wolfgang Ensinger, Salvador Mafe

**Affiliations:** † Departamento de Física de la Terra i Termodinàmica, Universitat de València, 46100 Burjassot, Spain; ‡ Departamento de Física Aplicada, 16774 Universitat Politécnica de València, 46022 València, Spain; § Materials Research Department, GSI Helmholtzzentrum für Schwerionenforschung, D-64291 Darmstadt, Germany; ∥ Department of Material- and Geo-Sciences, 26536 Technische Universität Darmstadt, D-64287 Darmstadt, Germany; ⊥ Allen Discovery Center at Tufts University, Medford, Massachusetts 02155, United States

## Abstract

Ionic flow nonlinear effects are helpful for sensing and signal processing in nanofluidic systems. Here, we develop a simple phenomenological model based on a distribution of Boltzmann-like electrical conductances to describe different forms of voltage-controlled negative differential resistance observed in charged conical nanopores. Multiple negative differential resistance phenomena show abrupt drops in the ionic current when the applied voltage exceeds a series of threshold voltages. We use the phenomenological model to describe the multiple states resulting from externally controlled salt precipitation at the conical pore tips. We consider both single- and multipore membranes, together with parallel and antiparallel arrangements of two membranes, as a function of the applied voltage, salt type, ionic concentration, and temperature.

## Graphical abstract

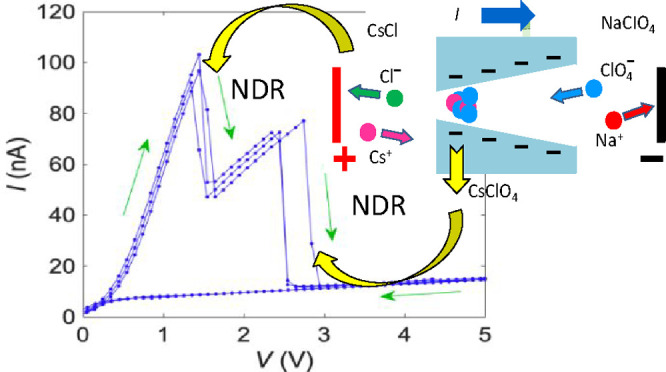

The external modulation of highly nonlinear effects in nanofluidic devices allows abrupt transitions in the ionic flow that are useful for sensing and actuating applications^1–6^ and neuromorphic computing.^7–17^ Here, we develop a simple phenomenological model based on a distribution of Boltzmann-like electrical conductances, which is capable of describing different forms of voltage-controlled negative differential resistance (NDR) observed in ionic systems, including those with charged, conical nanopores.^18^ Multiple negative differential resistance phenomena show abrupt drops in the ionic current when the applied voltage exceeds a series of threshold voltages. We use the phenomenological model to describe the multiple states resulting from the externally controlled salt precipitation at the conical pore tips. NDR effects have been employed in electronic solid-state switches and memories^19^ and have also been reported in different ionic liquid-state nanostructures.^20–30^ The external modulation of sequential multiple membrane conductance states should find immediate application in neuromorphic signal processing.^7–18^ In particular, it has been demonstrated that logical functions based on voltages and currents as input and output signals can be implemented using conventional electrochemical cells.^18,31,32^ Furthermore, the integration of nanofluidic units with multiple states and memories is central to the design of fluidic ionic circuits.^8–10,12–15,33–37^

The above applications can be guided by fundamental theoretical descriptions of the observed phenomena.^25,28,34,38–43^ In this study, the focus is on the multiple NDR and threshold voltage phenomena caused by controlled salt precipitation in single- and multipore membranes, including parallel and antiparallel arrangements. Salt precipitation at the nanoscale tip pore and then the threshold voltages follow a complex heterogeneous reaction that depends on the salt type and concentration, the pore size, shape, and surface charge, and the temperature, as described in ref 6. In order to explain the experimental facts observed, we employ a phenomenological model, based on a distribution of Boltzmann-like pore conductances and threshold voltages, in order to describe the different conductance states and NDR regions recently observed.^18^ We show that this simple model constitutes a useful tool to resolve and characterize the multiple conductances and state memories that provide nanofluidic NDR phenomena.

The nanopore characteristics have been described previously^18^ and are summarized here for the sake of completeness. The membranes have an exposed area of 1 cm^2^ and consist of conical pores obtained by irradiating a polyimide foil with single swift heavy ions.^44,45^ The number of pores can be approximately controlled during the irradiation process for membranes with low pore density, and the pore orientation is determined by the electric current direction and the left and right solution characteristics in the asymmetric track-etching process.^5,20,44^ The resulting tracks are subsequently functionalized by techniques, resulting in negatively charged pores with tip and base radii in the order of 10 and 100 nm, respectively.^45^ The pore surface charge is attributed to the ionization of the carboxylic acid moieties in aqueous salt solutions at neutral pH. The current (*I*)–voltage (*V*) curves are measured in an electrochemical cell confined in a double-shielded Faraday cage and mounted on an anti-vibration table. More details concerning the setup characteristics, the salt precipitation that leads to the NDR phenomena, and the experimental *I*–*V* and *I*–*t* (time) curves are given in Figure 1a–d.

**Figure 1:**
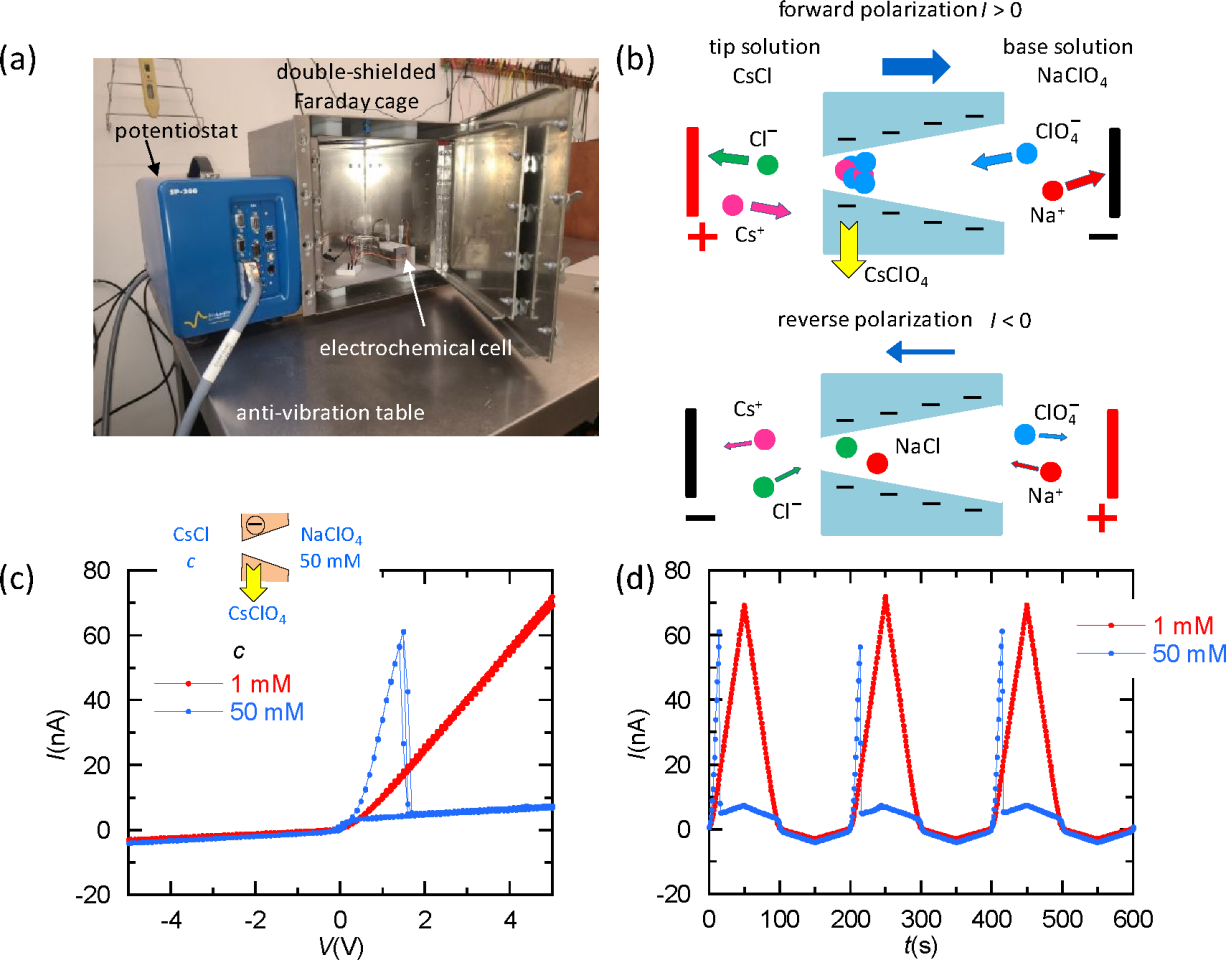
(a) Experimental setup. (b) Schematic representation of ionic transport through the conical nanopores. Ionic accumulation and pore blocking occur only at voltages of *V* > 0 (forward polarization), leading to salt precipitation. In contrast, ionic depletion occurs at *V* < 0 (reverse polarization) and gives a rectified current with no precipitation. (c) Experimental *I*–*V* curves for a single-pore membrane bathed by *c* = 1 and 50 mM CsCl (tip) and 50 mM NaClO_4_ (base) aqueous solutions. The significant current rectification arises from the conical charge distribution. The NDR effect caused by the ionic accumulation and CsClO_4_ precipitation at the pore tip occurs only for *c* = 50 mM. We considered up to 12 cycles under voltage sweeping, thus checking NDR reproducibility in longer term cycles. Typical deviations in the range of 10–15% were found. (d) Corresponding *I*–*t* traces for a driving triangular wave voltage with an amplitude of 5 V and scan rate of 50 mV/s.

Figure 1c and d shows the precipitation-induced NDR phenomena, characterized by the sharp current peak and subsequent drop that occur at the threshold voltage *V*_th_ > 0. This is followed by a quasi-ohmic low conductance region at *V* > *V*_th_. The fact that NDR phenomena are only observed at high enough salt concentrations suggests a solubility effect within the confined nanopore solution.^6,28,46^ Other possible NDR phenomena concerning electrode and interfacial membrane potentials could be discarded here because Nernst and Donnan potential drops are typically much lower than the threshold voltage: *V*_th_ is around 2 V in Figure 1c. Note also that, while nanobubble formation might also display some NDR features, these would result in rather erratic current readings, as opposed to the sharp response and reproducible behavior observed in Figure 1c and d. In addition, the observed salt type, ionic concentration, solution pH, and temperature-dependent solubility effects^6^ are all consistent with precipitation phenomena.

It is remarkable that, under the above NDR conditions, three usually winning strategies to increase the current *I* can become losing strategies instead:^6,11,18,20,28,29^ (i) Because the current increases steadily with the voltage *V* until *V* = *V*_th_, where it sharply decreases (NDR effect), the observed currents for *V* > *V*_th_ can be lower than those for *V* < *V*_th_, despite the increase in the driving force (see Figure 1c). (ii) Increasing the ionic concentration from *c*_1_ to *c*_2_ > *c*_1_ can give *I*_2_ < *I*_1_, despite the expected increase in the solution conductance due to the higher number of ionic carriers.^6,18^ (iii) For the case of retrograde salt solubility, increasing the temperature at *V* > *V*_th_ can give a current decrease despite the expected increase in the solution conductance due to the thermal activation of ionic carriers.^6^

Since no blocking and then no NDR effects are observed in the cases of negative voltages, where the small rectified current shows no significant salt precipitation, as well as in positive decreasing voltages, where the pore remains blocked until sufficiently small voltages are reached (Figure 1c), we will focus on the increasing positive voltage and precipitation onset of the experimental cycle only. We consider a parallel arrangement of *N* different pores of individual conductances *G*_*i*_ and currents *I*_*i*_ = *G*_*i*_ *V*, where *i* = 1, 2, ..., *N*. Because of the ionic accumulation at voltage *V* > 0, salt precipitation occurs at the threshold voltage *V*_th*i*_ for the *i*th pore, thus causing the NDR effect shown in Figure 1b and c. We tentatively describe this effect by introducing a voltage-gated pore probability *P*_*i*_(*V*) for the *i*th pore to be in the open state. At high enough voltages, significant salt precipitation will occur in all pores so that a small leakage conductance *G*_leak_ should be observed.

A minimal phenomenological model can then be based on *N* Boltzmann-like electrical conductances^47^ and a leakage quasi-steady-state current as follows:

1 $$I=\overset{N}{\underset{i=1}{\sum }}\left[G_{i}Q_{i}\left(V_{\mathrm{th}i}\right)P_{i}\left(V\right)+G_{\mathrm{leak}}\right]V,P_{i}\left(V\right)=1/\left\{1+B\exp\left[\left(V-V_{\mathrm{th}i}\right)/V_{\mathrm{thermal}}\right]\right\},V_{\mathrm{thermal}}=RT/F$$

In eq 1, the different threshold voltages *V*_th*i*_ may be distributed according to the function *Q*_*i*_(*V*_th*i*_), e.g., a Gaussian histogram^48,49^ for the distinct threshold voltages *V*_th*i*_ that could be reached along different cycles (see Figure 1c). However, for the sake of simplicity, we assume that *Q*_*i*_(*V*_th*i*_) = 1 in order to emphasize the sharp threshold character of the pore blocking. This limiting case could correspond to well-defined thresholds with small statistical deviations from the average values for the different experimental cycles used to establish *V*_th*i*_ (Figure 1c). In eq 1, *F*, *R*, and *T* are the Faraday’s constant, the gas constant, and the temperature, respectively, so that *V*_thermal_ = *RT*/*F* = 26 mV for *T* = 300 K in eq 1.

The conductances *G*_*i*_ and *G*_leak_ in eq 1 can be defined in terms of reference conductance *G*_ref_. They should depend on the pore radii and surface charge,^6^ together with experimental conditions, such as the ionic concentration, temperature, and salt solubility.^18^ In eq 1, *G*_*i*_ values define the different slopes of the *I*–*V* curve, *V*_th*i*_ values define the onset of the distinct NDR regions, and parameter *B* modulates the experimental current drops at these threshold voltages. The model is not as sensitive to parameter *B* as to parameters *G*_*i*_ and *V*_th*i*_. If we take *B* = 1 as a reference value, then *B* could be related to the ionic concentration, e.g., through Donnan interfacial potential *V*_Donnan_. In this case, *B* = exp­(−*V*_Donnan_/*V*_thermal_),^46^ and for a typical charged nanopore^6,47,50–53^ with *V*_Donnan_ = – 60 mV, *B* = 10 in eq 1.

We now give a survey of the typical results of experimental interest that can be obtained with eq 1. Note that, while the parameter values can be determined by comparing the model eq 1 to the experimental *I*–*V* curves, no attempt is made here to calculate those particular values that quantitatively reproduce these experimental *I*–*V* curves. Instead, we show that the observed data can be qualitatively explained by this simple model, which can be extended further for quantitative studies. Future studies could introduce experimentally motivated distributions *Q*_*i*_(*V*_th*i*_) on the basis of the track-etched nanopore radii^54^ and use more elaborated models to scale the single-pore blocking to the multipore membrane.^55^

Figure 2a shows the experimental *I*–*V* curve obtained for two single-pore membranes in a parallel arrangement with clear current peak resolutions. In this case, two NDR regions with different threshold potentials could be established by using distinct combinations of salts and concentrations.^18^ Figure 2b corresponds to the theoretical *I*–*V* curve obtained with eq 1 parametrically in *B*. Clearly, the model eq 1 allows the characterization of the two current peaks from the initial conductance slopes and threshold voltages, with a minor dependence on parameter *B*. Note also that because of the linear dependence of the slow driving signal *V*(*t*) with time *t* and the quasi-steady-state response of the current *I*(*t*) (Figure 1d), the theoretical *I*–*V* curve of Figure 2b would closely correspond to the *I*–*t* curve along a given time period for the case of increasing positive voltages, and it is not shown here. Figure 2a and b, together with Figure 1c and d, suggests new opportunities for multiple voltage-to-spike conversions using nanofluidic pores when the applied voltages exceed a sequence of threshold values. Also, the reproducible NDR phenomena should allow the amplification of small changes around the approximately defined threshold voltages (Figure 1c).

**Figure 2:**
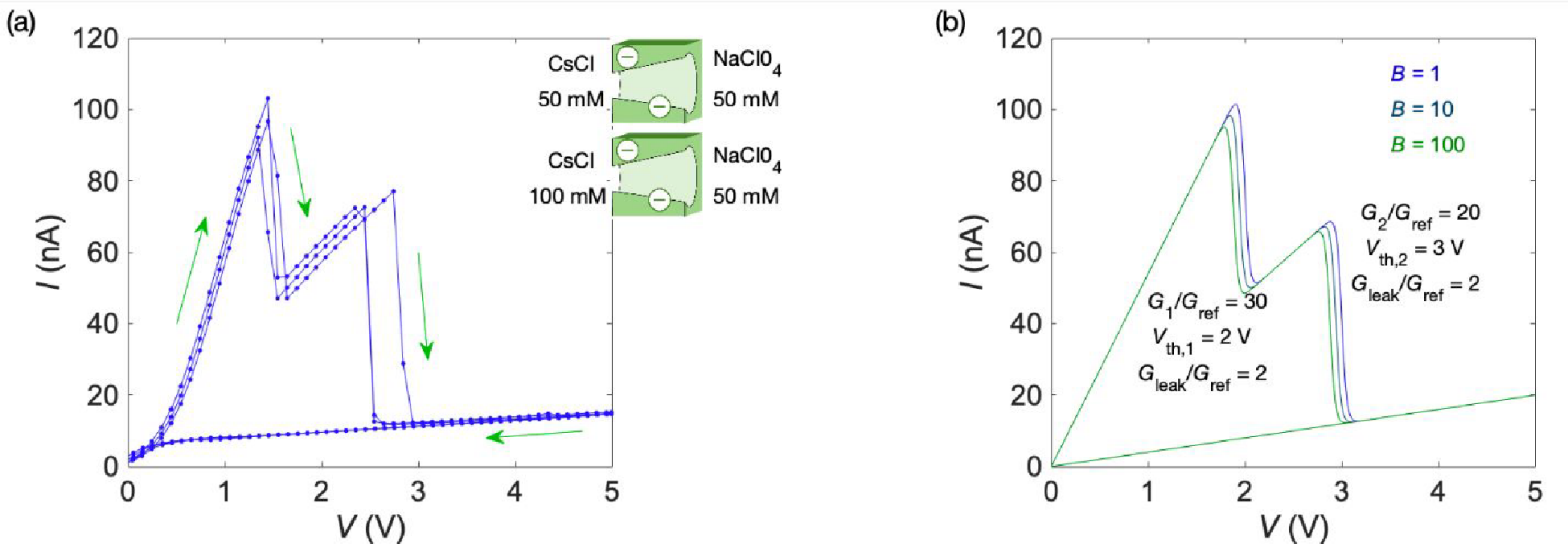
(a) Experimental *I*–*V* curve for a parallel arrangement of two single-pore membranes at the salt solution concentrations shown (inset).^18^ The use of different concentrations allows further separation of the threshold potentials, thus improving the resolution of current peaks. The NDR is due to the CsClO_4_ salt precipitation at the pore tips. The arrows indicate the directions of the applied voltage and the current hysteresis observed. (b) Theoretical *I*–*V* curve obtained with eq 1 and the model parameters shown (inset). Contrary to the cases of *G*_*i*_ and *V*_th*i*_, the curves are not highly sensitive to parameter *B*. Note that the two current peaks can be characterized by the initial conductance slopes *G*_*i*_/*G*_ref_ and the threshold voltage *V*_th*i*_, where *i* = 1 and 2. If we introduce the reference conductance *G*_ref_ = 1 nS, the current is given in nanoamperes.

Figure 3a shows the experimental *I*–*V* curve of a single membrane with a small number of conical pores instead of the parallel arrangement of two membranes shown in Figure 2a.^18^ Multipore membranes can also exhibit multiple threshold voltages due to the different pores that are sequentially switched to low conductance states upon salt precipitation at the pore tips. The limited separation obtained between the successive NDR thresholds arises from multiple pores that are progressively blocked at increasing voltages. In this case, the intrinsically noisy precipitation effects, which are more pronounced in multipore membranes, provide identification of the conductance states. Remarkably, the theoretical *I*–*V* curve of Figure 3b suggests that eq 1 is still able to resolve and characterize four current peaks in terms of the initial conductance slopes *G*_*i*_/*G*_ref_ and threshold voltages *V*_th*i*_. However, threshold voltage sequences could not be accurately resolved when the membrane has a high number of pores, leading to mixed current peaks.^18^

**Figure 3:**
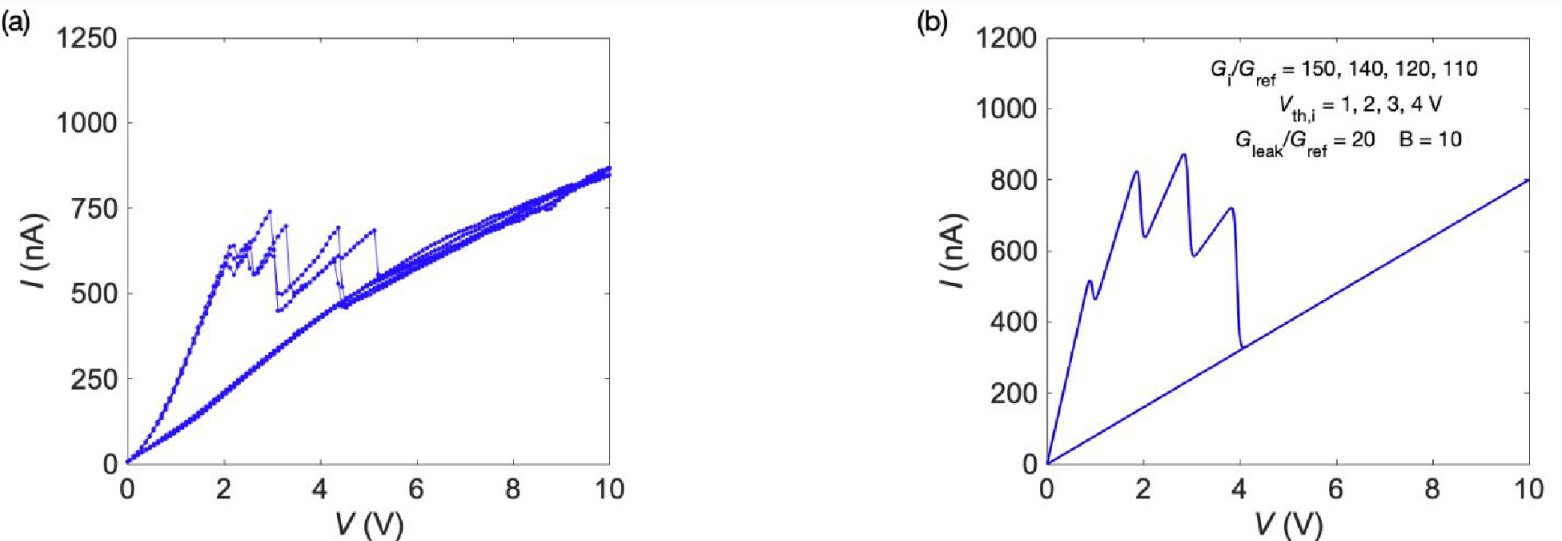
(a) Experimental *I*–*V* curve for a multipore membrane with a small number of conical pores bathed by the salt solutions 50 mM CaCl_2_ (pore tip) and 100 mM Na_2_SO_4_ (pore base).^18^ The observed current peaks and drops arise from CaSO_4_ salt precipitation at the pore tips. These peaks may give a rough estimation of the different pores that are sequentially blocked at distinct voltages. Note, however, that additional wider pores or those with a geometry making precipitation more difficult could also be present in the membrane. The different NDR thresholds correspond to the multiple pores that are sequentially blocked at distinct voltages. (b) Theoretical *I*–*V* curve obtained with eq 1 and the model parameters shown (inset). The four current peaks resolved can also be characterized by the initial conductance slopes *G*_*i*_/*G*_ref_ and the threshold voltages *V*_th*i*_, where *i* = 1, 2, 3, and 4.

In addition to the case of the multiple pores, threshold voltages, and conductance states of Figures 2 and 3, Figure 4a considers the effect of the temperature on the *I*–*V* curve of a single-pore membrane.^6^ Here, the NDR is caused by Ca­(OH)_2_, and as opposed to most pairs of salts, the retrograde solubility of this salt makes the precipitation increase with the temperature. The threshold potential shift in Figure 4a clearly shows this effect. Initially, the conductance slope before blocking increases with temperature *T* due to the higher thermal activation of the ionic carriers in the pore solution. Eventually, however, this temperature effect is reversed because of the decreased solubility of the precipitated salt at a high temperature. Again, the model eq 1 can qualitatively reproduce this behavior in terms of the pore conductances and threshold potentials of Figure 4b. Note that the usual case of non-retrograde solubility could also be accounted for theoretically by increasing the threshold voltages rather than decreasing them with the temperature.^18^ Taken together, these results suggest that, in addition to the salt type, ionic concentration, and applied voltage (Figures 1–3),^6^ the temperature constitutes an additional control parameter for the multiple threshold voltages and conductance states.

**Figure 4:**
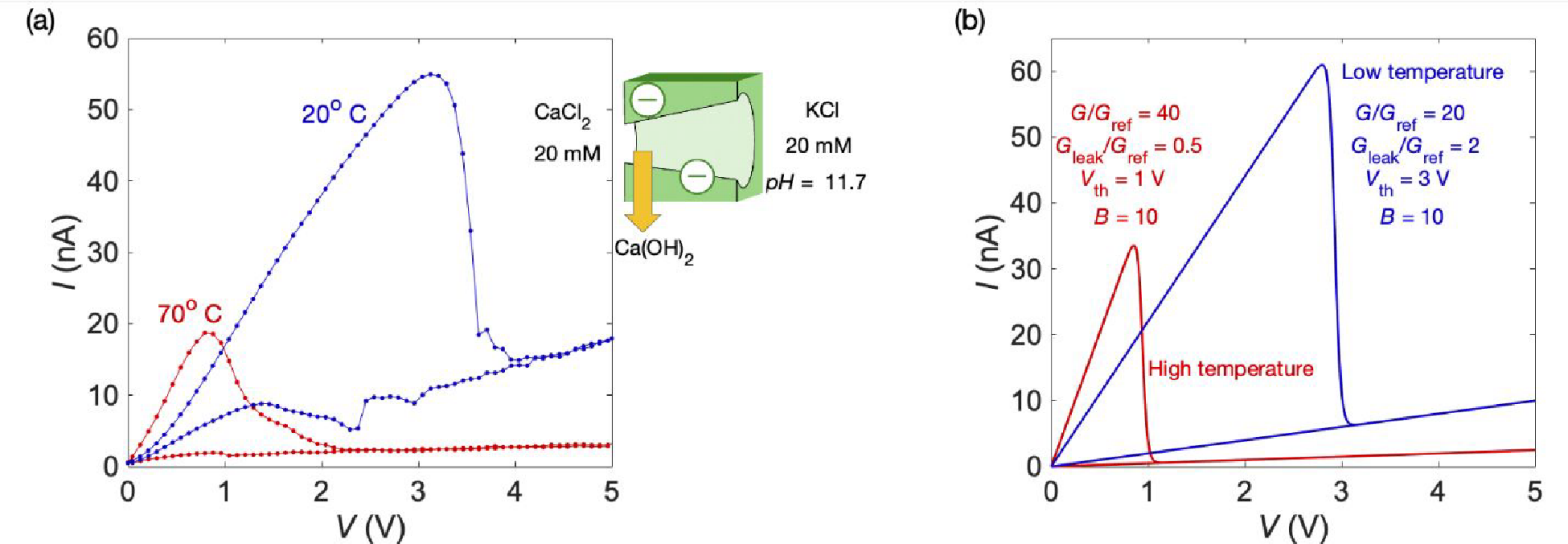
(a) Experimental *I*–*V* curves obtained at different temperatures for a single-pore membrane bathed by the salt concentrations and solution pH values shown (inset).^18^ The NDR is caused here by Ca­(OH)_2_ precipitation at the pore tip. Note that this particular salt exhibits retrograde solubility, which decreases with the temperature, as indicated by the observed threshold potential shift. (b) Theoretical *I*–*V* curves obtained with eq 1 show that the current peaks can also be characterized by the conductances and threshold voltages shown (inset).

In a different context of high potential applicability, Figure 5a shows a digital-like response obtained with an antiparallel arrangement of two single-pore membranes.^18^ The sharply defined voltage thresholds and conductance states allow the establishment of a set of logical responses, including reversible logical functions, which should be of practical value for the implementation of multiple-state memories and multivalued input/output nanofluidic functionalities.^18,37^ In addition, the comparison of Figure 2 with Figure 5 suggests that the correlation between threshold potentials and ionic concentrations can be used in sensing applications.^6^ In this case, the model results of Figure 5b can also be helpful to characterize the slope conductances and threshold voltages theoretically.

**Figure 5:**
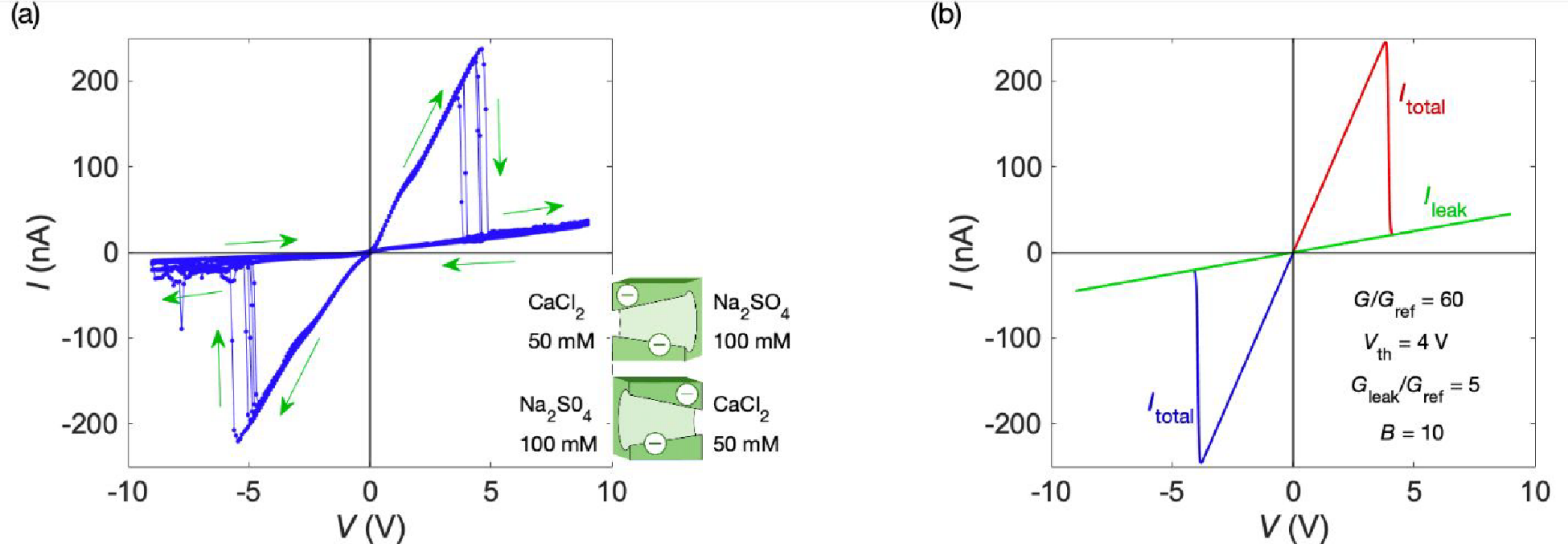
(a) Experimental *I*–*V* curve for the case of two single-pore membranes arranged in antiparallel configuration and the salt solutions shown (inset).^18^ In this case, the positive and negative threshold voltages give a digital-like response with a non-pinched behavior at zero voltage. The salt electrolytes used in the two pores are different to show the salt precipitation phenomena with different solutions and pores. (b) Theoretical *I*–*V* curves obtained with eq 1 and the model parameters shown (inset).

Threshold voltages can be externally controlled by the salt type and concentration, pore size, shape, and surface charge, and temperature.^6^ Although NDR threshold voltages show some stochastic behavior due to their nanoscale spatial scale, the experiments in Figures 1c, 2a, and 5a demonstrate that reliable average values can still be defined. A minimal phenomenological model based on the distributions of Boltzmann-like electrical conductances permits the theoretical description of the multiple threshold voltages and membrane conductance states obtained from externally controlled salt precipitations at conical pore tips. Since sensing and actuating circuits can use NDR to detect/amplify small changes in an ionic solution as well as to mimic neuron-like spiking behavior, we believe that this coarse-graining model is a simple initial step, and more detailed theoretical approaches will be developed. Also, because of the additive characteristics of the pore currents,^18,36^ the parallel and antiparallel arrangements of two membranes should be helpful in nanofluidic circuitry. In particular, heterogeneous threshold ensembles can be useful for the signal processing of weak signals^49,50^ and the system design of decision trees.^56^ Taken together, the broad range of effects described concerns voltage, salt type, ionic concentration, temperature, and membrane configuration, thus suggesting new opportunities for sensing and actuating using conventional electrochemical cells.

## Data Availability

Data will be made available under reasonable request.
